# “We’re, Like, the Most Unhealthy People in the Country”: Using an Equity Lens to Reduce Barriers to Healthy Food Access in Rural Appalachia

**DOI:** 10.5888/pcd17.200340

**Published:** 2020-12-24

**Authors:** Kathryn Cardarelli, Emily DeWitt, Rachel Gillespie, Heather Norman-Burgdolf, Natalie Jones, Janet Tietyen Mullins

**Affiliations:** 1 Department of Health, Behavior & Society, University of Kentucky, Lexington, Kentucky; 2 Family and Consumer Sciences Extension, University of Kentucky, Lexington, Kentucky; 3 Department of Dietetics and Human Nutrition, University of Kentucky, Lexington, Kentucky

## Abstract

**Introduction:**

Obesity disproportionately affects rural communities, and Appalachia has some of the highest obesity rates in the nation. Successful policy, systems, and environmental (PSE) interventions to reduce obesity must reflect the circumstances of the population. We used a health equity lens to identify barriers and facilitators for healthy food access in Martin County, Kentucky, to design interventions responsive to social, cultural, and historical contexts.

**Methods:**

We conducted 5 focus groups in Martin County, Kentucky, in fall 2019 to obtain perspectives on the local food system and gauge acceptability of PSE interventions. We used grounded theory to identify perceived barriers and facilitators for healthy eating.

**Results:**

Thirty-four adults (27 women; median age, 46 years) participated in 5 groups. One prominent theme was declining interest in farming; many participants believed this decline was generational. One participant noted, “Most of my adult male relatives worked in the coal mines, and they worked 6 days a week. . . . My grandpa had the garden, but then my dad’s generation is the one quit gardening.” Another shared, “You would probably have to have someone to teach [gardening].” Instead of enhancing farmers markets, participants suggested building community capacity for home gardens to increase vegetable consumption.

**Conclusion:**

Our findings demonstrate the importance of obtaining community input on the development of PSE interventions to mitigate inequities in obesity. Although farmers market interventions were deemed not feasible, other solutions to enhance access to produce were identified. Developers of community-responsive PSE interventions to improve healthy eating in rural, food-insecure locations should consider using an equity-oriented prevention framework to ensure acceptable interventions.

**Summary**

**What is already known on this topic?**

The prevalence of obesity is disproportionately high among people living in rural areas, yet many policy, systems, and environmental interventions designed to improve healthy food access in these environments have not been successful.

**What is added by this report?**

An equity-oriented obesity prevention framework can guide investigators in identifying or tailoring acceptable interventions unique to a community’s needs.

**What are the implications for public health practice?**

Community input to intervention development is crucial to the success of environmental changes to expand healthy food access in rural areas.

## Introduction

Rural communities in the United States have disproportionately higher rates of preventable obesity-related illness and death compared with their urban counterparts (1). Characteristics of some rural regions, such as Appalachia, present challenges that exacerbate the high rates of obesity and related health conditions in certain populations (2,3). The lack of reliable food retailers in Appalachia reflects a malfunctioning food system unable to support healthy eating patterns (4). In addition, persistent poverty and unemployment are linked to a high prevalence of preventable mortality in Appalachia (2,5).

Social, political, and historical contexts influence the effectiveness of programs and interventions aimed at promoting healthy food choices (6). These contexts are unique to each community, with distinctive regional characteristics among Appalachian communities (7). Policy, systems, and environmental (PSE) interventions and strategies designed for communities with a disproportionately high prevalence of obesity, such as communities in Appalachia, are needed. However, established approaches have been largely ineffective in adult populations that have inequities (8); therefore, new and novel frameworks for designing and implementing successful, equitable interventions are necessary.

The Getting to Equity (GTE) framework provides a guide for implementing obesity prevention activities that gives priority to health equity principles (9,10), an approach that is potentially important in Appalachia (Figure). Each quadrant in the framework represents a type of intervention approach. The upper 2 quadrants, which include increasing healthy options and reducing deterrents, focus on potential policy-change and systems-change interventions. The lower 2 quadrants, which include building on community capacity and improving social and economic resources, reflect individual and community resources and capacity. Each identified strategy in each quadrant has shown promise or relevance in the mitigation of health disparities. Kumayika argues that balance and synergy are needed among the strategies (4 quadrants) to be effective at producing sustainable, positive change (10).

**Figure:**
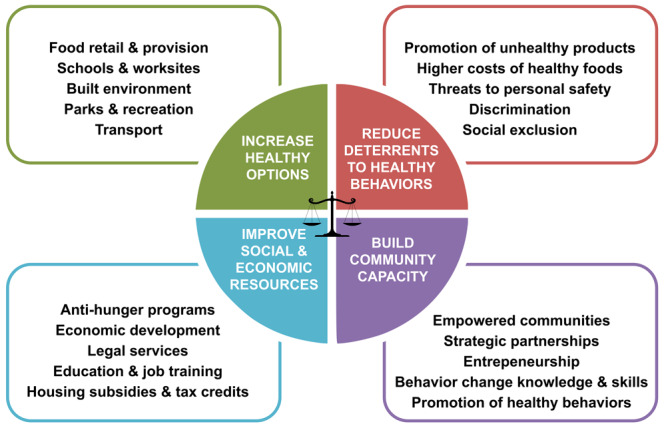
Getting to Equity framework for obesity prevention. Source: Kumanyika (9). Reprinted with permission from the National Academy of Sciences, Courtesy of the National Academies Press, Washington, DC.

Our study, in Martin County, Kentucky, was part of the larger, multiyear High Obesity Program, which has the overall aim of reducing rural obesity and decreasing the risk of preventable mortality (11). Although the High Obesity Program is multifaceted, it emphasizes increasing geographic or financial access to healthy foods. In addition, the High Obesity Program requires use of existing infrastructure in rural communities, such as the Cooperative Extension Service and community coalitions. The aim of this study was to use the GTE framework to identify barriers to and solutions for increasing access to healthy foods in a rural, resource-poor environment.

## Methods

We conducted our focus group study in September and October 2019 in Martin County, in eastern Kentucky, which is adjacent to West Virginia. Approximately 39% of residents live in poverty, and the county struggles with high unemployment (12.4%) and outmigration (a 13.4% reduction in population from April 2010 to July 2019) (12). According to the Food Access Research Atlas, more than 33% of county residents live 20 miles or more from the nearest supermarket, which would classify the entire community as a food desert (13). Approximately 1 in 5 Martin County households are considered food insecure (14). One of the few community assets to promote healthy eating in the county is the nonprofit organization Grow Appalachia. Established at Berea College in 2009, the mission of Grow Appalachia is to increase access to fresh fruits and vegetables by building capacity to successfully grow home gardens. Grow Appalachia is active in Martin County, supplying participants with assistance to grow food (15).

In summer 2019, we purposively recruited adults from Martin County for participation in focus groups. The Martin County Extension agent recruited participants, as did community coalition members. We placed informational flyers in the Martin County Extension Office and posted information on its Facebook page. Eligibility criteria for participation were being 18 or older, speaking English, and residing in Martin County. Participants completed written informed consent and completed a brief sociodemographic survey. Participant assignment to focus groups was random with 1 exception: staff members of a local middle school were recruited to participate in a focus group held at that location. A trained moderator facilitated the focus groups (K.M.C.) using a written moderator guide (Box), and 2 research team members took notes (E.D., R.G.). All focus groups took place in September and October either in the Martin County Extension Office or in the local middle school and lasted approximately 1 hour. Participants received a $25 voucher for a local grocery store as an incentive to participate. The University of Kentucky Institutional Review Board approved this study.

**Box. Questions for Focus Groups on Healthy Eating in Appalachia**

**Where are the places you can purchase food in your community?**

• How easy it is to get fruits and vegetables at these locations?

• Do many people in your community purchase food at farmers markets?

• Where can people go in your community to get food if they are unable to purchase it? (eg, food pantries, churches)

**Do you think your community is designed to promote healthy eating choices? Why or why not?**

• What factors in your community make it easier to eat healthy?

• What factors in your community make it harder to eat healthy?

• Would you consider transportation a barrier?

**What other resources do you think would be helpful to have in your community to allow people to purchase fruits and vegetables?**

**What would be some ways to motivate or encourage people in your community to eat fruits and vegetables?**

(Bullet points refer to probes the moderator could use for further discussion, if needed.)

We summarized the data from the brief sociodemographic survey, and we compared the sociodemographic composition of focus group participants with the composition of the Martin County population as reflected by data from the US Census Bureau (12). Focus groups discussions were audio recorded and transcribed verbatim. Multiple investigators reviewed focus group transcripts using a grounded theory approach (16). Investigators used an iterative inductive–deductive approach to identify themes on assets and barriers to healthy eating in the community. These themes formed the basis of codes that were analyzed in NVivo software version 12 (QSR International). Investigators then used the GTE framework to categorize themes according to the 4 quadrants of intervention approaches and selected illustrative quotes for each theme. We conducted this analysis during January–March 2020.

## Results

Thirty-four adults participated in 5 focus groups. The median age of participants was 46 years, and 27 were women (Table 1). All participants were non-Hispanic White, and most participants had some college education or were college graduates. Compared with the Martin County general population, study participants were less racially/ethnically diverse, slightly older, and had higher levels of education.

**Table 1:** Sociodemographic Characteristics of Focus Group Participants (N = 34) and the General Population of Martin County, Kentucky, 2019

Characteristic	No. (%)	Martin County, %^a^
**Age, median, y**	46	39
**Sex**		
Female	27 (79)	45
Male	7 (21)	55
**Race**		
Non-Hispanic White	34 (100)	92
Non-Hispanic Black	0	7
Other races combined	0	1
**Hispanic ethnicity**	0	3
**Education**		
<High school graduate	1 (3)	26
High school graduate	4 (12)	39
Some college	12 (35)	25
College graduate	17 (50)	9
**Household income, $**		
<20,000	8 (24)	—b
21,000–59,999	13 (38)	—b
≥60,000	13 (38)	—b

a Data source: US Census Bureau (12).

b No analogous data categories available from the US Census Bureau.

Investigators established several independent but interconnected themes related to healthy eating. Participants identified myriad barriers to healthy eating (Table 2) and a smaller number of assets in the community that promote healthy eating. These assets included Grow Appalachia and Cooperative Extension Service programming, both of which address barriers identified by participants to growing food, including knowledge of how to grow a garden and the ability to grow and sell food for a profit. Deep-rooted community pride was also made evident as an asset. These assets collectively lie within the GTE quadrant of building community capacity. Several participants drew connections between Grow Appalachia and their capacity to grow and consume produce year-round.

**Table 2:** Barriers to Healthy Eating in Martin County, Kentucky, as Identified by Focus Group Participants and Organized Within the Getting to Equity Framework^a^

GTE Quadrant and Participant Narratives	Illustrative Quote^b^
**GTE quadrant: Increase healthy options**	
Limited food retail options	[O]ne of the main problems with [local grocery store] is not enough people in our community buy the fruits and vegetables, and so they don’t keep as much on hand because it doesn’t sell as quickly here.
Lack of access to produce	I know for the senior citizens, like, we will order bananas but we can’t get them around here ‘cause they don’t have enough for us to go purchase. So we have to order them and they come frozen. And when you open it up, it’s black.
	We do have a local produce, private owned produce store, but they don’t keep a lot of stuff.
**GTE quadrant: Reduce deterrents to healthy behaviors**	
Cost of healthy food	Fresh fruits and vegetables are not cheap.
	A lot of people are on fixed income . . . and it’s hard to eat healthy . . . it’s the bottom line. It is way expensive to eat healthy.
Availability of fast food	It’s like, say you go to McDonald’s or Wendy’s or somewhere, you know a salad is $4 or $5 compared to you know, chicken nuggets a dollar.
	You can go out and get a dollar hamburger versus $5 for fruit.
	You can buy a box of Little Debbie’s for $1.99 and you can’t buy hardly anything out of the produce case for $1.99.
	I am sure there are a lot of kids out there right now that’s in high school that have very little fresh vegetables their whole life. Their parents have always went to McDonalds or a pizza place.
	I think it’s just tradition, people are used to eating their fatty . . . fried foods. . . . I would agree with that. I think it’s just part of the culture. That’s just what we’re used to.
Transportation barriers	Transportation is a very big issue . . . it’s getting out there and getting them to a grocery store that’s a barrier for them.
	Transportation is the biggest issue for this community. . . . It is a big obstacle. . . . It is getting them to church, it is for getting them to school, it is for getting them to the grocery store, to the doctor, it is just a major issue.
	I have people that pay people to drive them out of the hollow basically.
**GTE quadrant: improve social and economic resources**	
Persistent poverty	I mean, we never knew we were poor until Johnson and Kennedy came and told us we were poor.
	Because they are not going to ask. I think it is just a pride thing for some people.
	Honestly, my biggest thing is that I can take an elderly woman who lives alone and is a widow and she gets $15 a month in food stamps. And I think that is insanity. She gets no food vouchers — she living off $771 a month.
	I mean, we’re, like, the most unhealthy people in the country. This part, I mean that’s just honest, central Appalachia it is.
**GTE quadrant: build community capacity^c^**	
Lack of cooking skills	There is a whole generation just like me . . . that is something that we didn’t do, so we don’t even know how to teach our kids to do that. There is a whole gap there of you know.
	They are some of the younger generation that asks, “Dad, well, how do you fix corn, how do you fix green beans?” They don’t know how. They don’t know to put it in a pot, put some water in it and put it on boil . . . they have no clue how to fix fresh vegetables.
	When RAMP [local food pantry] gives out produce, we have suppliers that send us stuff like eggplant and squash. Stuff that I have never heard of and can’t pronounce and stuff like that. And people don’t want it.
Lack of interest in farming	There’s no money in it . . . for the work and time and effort you put into it, if you don’t just enjoy doing it, there is no money in it. . . . You can’t do it and make your car payment every month. You couldn’t use it as a second income. There is no way to be profitable with it.
	It is a good thing if kids get to see it made . . . or get to see it grown, or whatever. And they know where, my grandkids don’t know where stuff comes from. They don’t work in a garden.
	You would probably have to have someone to teach people because while there aren’t any farmers in the county, they’re getting old or they have already died off and heaven forbid the kids would ever have to work in a garden.
	Most of my adult male relatives worked in the coal mines and they worked 6 days a week. . . . My dad left before sunrise and home after dark. Between coaching my little league and fishing.
	That whole generation of working people were worked their fingers to the bone.
	My grandpa had the garden, but then my dad’s generation is the one quit gardening.

a The Getting to Equity framework provides a guide for implementing obesity prevention activities that gives priority to health equity principles (9,10).

b Selected qualifying quotes included; not all quotes included per GTE framework and qualitative methodology.

c Assets (Grow Appalachia, community pride, and Cooperative Extension Programming) identified by participants would be categorized into this quadrant, but they are not included here.

Where I was in the Grow Appalachia project, they paid for all my seeds and everything. . . . I bet there was between tools and everything, well over a $1,000 put into my garden.

I was a participant in [Grow Appalachia], and I enjoyed it. . . I already knew a lot, but I have learned a lot more about canning and different things . . . we grew tomatoes, cucumbers, green beans, corn, zucchini, squash . . . peppers.

[Referring to Grow Appalachia] What helped me most from that program was, um, my husband passed away 3 years ago, and since then it’s been really hard to get it plowed. I have a plow, but it’s big and I can’t operate it. . . . That was so helpful to me, to get it plowed that first time.

Because of community support from programs like Grow Appalachia, participants expressed the idea that residents could grow their own produce for consumption. Participants also described a distribution network that existed across the community in which residents shared produce with neighbors and family members, rather than selling it.

I do share. I’ve not sold anything this year; it was the first year I had that big a garden. But yeah, my grandma, my parents, whoever, they want to drive out and help. I told them if they want to come help pick it, they can have some.

Yeah, I can answer that for myself there. When I raise things, I mean, I don’t sell it. I don’t believe in selling it. If I have got, usually I got a whole bunch, I give it away.

I know when I had a garden, and I had extra produce, I would tell people you can have anything you want they just have to come get it.

Participants revealed a keen awareness of the decline in the local farmers market. They connected the decline to generational shifts in career opportunities. As coal mining gained popularity in the region, people prioritized mining over farming.

Most of my adult male relatives worked in the coal mines, and they worked 6 days a week. My dad left before sunrise and home after dark. . . . My grandpa had the garden, but then my dad’s generation is the one that quit gardening.

Moreover, although a clear desire for homegrown produce was apparent among community members, the lack of interest in farming may result from the local view that cultivating homegrown produce is labor-intensive. Participants indicated that farming is not a lucrative endeavor in this region, further deterring interest among this population. Thus, the farmers market continues to dwindle in this county because of a lack of participating growers.

Dad sells at the farmers market, and he has noticed it seems to be declining a little bit, especially as the year goes on. It starts out pretty strong, he says, but as the year goes on. . . . I don’t know if they get burned out on produce, everyone gets used to eating fast food and stuff.

There’s no money in it. For the work and time and effort you put into it, if you don’t just enjoy doing it, there is no money in it. You can’t do it and make your car payment every month. You couldn’t use it as a second income. There is no way to be profitable with it. Unless you are doing it on a mass scale.

Participants described opportunities for encouraging homegrown produce, including enhanced knowledge of food preservation and opportunities to learn from those who have become experts through practice; however, most participants perceived opportunities as limited in their community.

But it was, like, a couple in my church that does that stuff, and they kind of walked me though it and showed me. And I just wish we had more resources to show us how to do those things.

Like our garden, I think I would plant a lot more, if I knew more about how to do the canning.

Yeah, you know, he’ll have, you know, lots of, you know, a lot of people have corn. Corn, you know, I’m pretty sure everybody has corn normally certain times of year, but green beans too quick. And you know, he always has lots of squash, and cucumber, tomatoes and stuff like that, and packs it up and takes it all home.

We like a certain thing, we want cucumbers, and we want green beans, and we want tomatoes, and my kids don’t really look at nothing else when we come. So, like you said, more green beans please.

Although preferences were established, participants described being motivated to make healthy choices to set an example for younger generations.

## Discussion

Using the GTE framework for obesity prevention, our study identified many barriers to, and a smaller number of solutions for, increasing access to healthy foods in the Appalachian region of Kentucky. Applying an equity-oriented lens to understanding rural food access requires recognition of fundamental conditions that shape individual experiences and the rejection of biases that blame individuals for circumstances beyond their control (10). Our findings reflect the decline of farming as an occupation in rural Appalachian communities, yet many participants spoke of home gardening as a self-sustaining food source for themselves or a network of people, such as family members or neighbors. Garden produce unused by the grower, we learned, is distributed to the community through an informal economy of food bartering and sharing. Food, in this fashion, acts as its fundamental purpose, a commodity valued at a worth woven into the fabric of Appalachian culture. This concept is important to consider when designing PSE interventions focused on food access in Appalachia.

The declining fiscal contribution of farming, as well as the practice itself, has been gradual yet consistent in Appalachia (17). As our findings suggest, the decline in farming could be attributed to generational shifts in industry opportunities. In Appalachia, farming practices began to deteriorate in the late 19th century, when a new economic stimulus appeared in the form of timbering and coal mining (18). Since then, the region has continued to experience agrarian decline. The 2017 Census of Agriculture for Martin County showed 30 farms and 43 total producers (60% male, 40% female); the average age of producers was 47. Ten farmers reported being younger than 35; 17 reported farming as their primary occupation, and only 3 farmers sold directly to consumers (19). Furthermore, the Kentucky Appalachian region lost a disproportionate amount of farmland from 2007 through 2012: 9.2% compared with 0.8% across the United States (17). The effect of these declines in Appalachia has yet to be fully explored. However, it begs further investigation when considering factors that have led to the persistent poverty levels, poor health status, and dissolved food access points in this community.

Health disparities in Appalachia, including those related to continued outmigration, have led to economic decline and increased poverty (20). From 2010 to 2019 alone, the population in Martin County decreased by an estimated 13.4% (12). The GTE framework further guides synergetic interventions and explores the intertwining realms that influence equity in the context of outmigration, economic decline, and increased poverty. Therefore, it is worth continuing to investigate the chasm between a community practice of food sharing and a farming decline as a mode to incorporate GTE principles to improve healthy food access in rural Appalachian communities such as Martin County.

The shift from traditional farmers markets is increasingly evident, leaving communities and food systems to envision alternative modes in which to implement healthier lifestyle behaviors, including fruit and vegetable consumption (21). Small farms and home gardens are important assets in Appalachian heritage; they have numerous social and historical implications and reflect strong local values, such as self-sufficiency and esteemed locavore practices (sourcing and consumption of locally grown or produced foods), bolstering their feasibility as effective interventions (22). The findings from our focus groups echo the role of small-scale home gardens in this Appalachian community as a mode of increasing access to fresh fruits and vegetables. Appalachian communities value these cultural customs, as evidenced by the rich history of heirloom vegetable seeds in the region (22). Future work should use culturally relevant tools and examine the existing food system infrastructure when developing novel strategies to increase access to fruits and vegetables outside traditional approaches. Although farmers markets have been viable interventions in some communities (23), they may not be suitable solutions for all, given the unique characteristics of Appalachian communities. For example, a qualitative study of 15 low-income Appalachian residents found that only 1 person regularly visited a farmers market, citing pricing and inconvenience as barriers (24). Although respondents reported generally positive attitudes toward farmers markets, the economic and cultural environmental landscapes and other barriers do not make them a plausible intervention for all Appalachian communities (25,26).

The findings from our focus groups add to the growing body of research illuminating the health inequities Appalachian communities face. It is important to note the rapid decline of the socioeconomic landscape in rural communities compared with their urban counterparts (27). Although common barriers, such as affordability and access to healthy food, exist among low-income residents of both rural and urban communities, Appalachia has unique challenges, including low population density, geographic isolation, and persistent poverty, that amplify these barriers (7,25). An increase in poverty leads to less food affordability, particularly among rural low-income populations in the Appalachian region (27). Additionally, since the completion of our focus groups, 1 of only 3 grocery stores in this community closed. This further reinforced the food access barriers in this community.

Inadequate access to healthy foods contributes to the declining health status of rural communities, including increased rates of obesity and chronic diseases (1,3). Inadequate access to healthy foods is challenging when coupled with aforementioned barriers and transportation access. Collectively, these factors make rural Appalachian communities distinctly different from impoverished urban communities when addressing improvements to food accessibility and, more broadly, the health status of populations. Despite probing feasible solutions for the multitude of barriers their food system presented, participants were not forthcoming with many solutions aside from suggested enhancement to current practices such as home gardening.

For interventions to be successful, they must be tailored to unique community needs. For example, participants in our study deemed farmers markets impractical, although they are a common intervention to mitigate problems with food systems in rural communities. However, participants identified some community assets, particularly Grow Appalachia, an initiative established to address food insecurity by working with families to grow produce at home. Through training and technical assistance, Grow Appalachia enables communities to prepare, plant, and cultivate home gardens, improving access to nutritious foods and enhancing social enterprise to sustain an equitable food system (14). In 2019, the Martin County Cooperative Extension Office partnered with Grow Appalachia to enhance food security. The partnership enables Grow Appalachia to provide home gardeners with resources and services, such as equipment and seeds, while the Cooperative Extension Service provides ongoing support and training throughout the growing season. By supporting individual gardeners, the Grow Appalachia framework may be more effective in improving access to fruits and vegetables than sustaining the farmers market in this rural community. Furthermore, because of coronavirus disease 2019 (COVID-19), interest in the victory garden toolkit on how to grow gardens — distributed by Cooperative Extension offices — has increased. The increased interest lends support for continued interventions that focus on home gardening. Food preservation and cooking classes are additional services that support home gardeners and promote healthy eating (28) and are services identified as desirable to this community.

Future initiatives must consider the deeper roots of systemic issues to implement effective and equitable solutions. One issue influencing food choice in this community is basic food security. Martin County has historically faced high rates of food insecurity. Yet, because of the COVID-19 crisis, food insecurity is projected to increase by more than 5% to 26%; 1 in 4 households will experience food insecurity in the years to come (29). The repercussions of food insecurity will be numerous for an already vulnerable population. Moreover, Appalachia experiences persistent poverty (16.3% vs. 14.6% for United States), with Appalachian Kentucky having the highest poverty rate among all states in the Appalachian region (25.6%) (26). To address food access inequities, poverty and food security status must first be addressed. Addressing only 1 quadrant of the GTE framework is likely insufficient to implement sustainable change in food access. The incorporation of additional strategies that support the 3 remaining quadrants of the GTE framework are needed to balance and enhance effectiveness and sustainability of future interventions. Furthermore, finding culturally relevant facilitators to promote healthy choices will be key to behavior change.

Our study has several limitations. We did not randomly select our sample; we used a purposive, community-engaged approach to recruiting. Participants reported higher levels of education than the general county population. Additionally, our sample included more women than men and older participants (13), limiting the external validity of our findings to other rural or Appalachian populations. In an equity perspective, this is an important limitation and suggests that the barriers identified in our study are likely not the only barriers that impede access to healthy food in the community. Finally, social desirability bias may have influenced respondents’ comments. Despite these limitations, our study demonstrates the value of framing barriers to food access in a rural Appalachian population with an equity lens. Future PSE interventions to address food access in this and similar populations should consider using the GTE framework to envision new approaches that explicitly acknowledge social inequities that challenge healthy eating.

Few macro-scale approaches, such as enhancing farmers markets, have shown broad success in rural Appalachia, which speaks to the heterogeneity of these communities (24,30). Designing food access interventions in rural Appalachia that explicitly acknowledge the social inequities in the region and actively engage community members are likely to be more successful than those that do not. This study revealed a novel overarching theme: enhancing community capacity through various channels that depend on the existing resources reported by community residents. Our findings validated the importance of having community buy-in to support the small grower through multiple avenues, including Grow Appalachia and Cooperative Extension Service programming. The COVID-19 pandemic has further affected the food system in Appalachian communities. Instead of enhancing farmers markets, future investigators focused on obesity prevention work in rural Appalachia must learn about the local food system and culture. This focus will enhance community capacity for growing personal gardens, increase food access availability, and improve equity.

## References

[1] Befort CA , Nazir N , Perri MG . Prevalence of obesity among adults from rural and urban areas of the United States: findings from NHANES (2005–2008). J Rural Health 2012;28(4):392–7. 10.1111/j.1748-0361.2012.00411.x 23083085 PMC3481194

[2] Lane NM , Holmes GM , Arcury TA , Schwalbe ML , Randolph R , Frank J , Exploring bright spots in Appalachian health: case studies. 2018. https://www.arc.gov/report/identifying-bright-spots-in-appalachian-health-statistical-analysis-2. Accessed November 17, 2020.

[3] Slack T , Myers CA , Martin CK , Heymsfield SB . The geographic concentration of US adult obesity prevalence and associated social, economic, and environmental factors. Obesity (Silver Spring) 2014;22(3):868–74. 10.1002/oby.20502 23630100 PMC13722265

[4] Booth J , Wei K , Little A . Examining the impact of food environment changes on county-level obesity prevalence in the Appalachian region. J Health Dispar Res Pract 2017;10(4):14–33.

[5] Kiang MV , Krieger N , Buckee CO , Onnela JP , Chen JT . Decomposition of the US black/white inequality in premature mortality, 2010–2015: an observational study. BMJ Open 2019;9(11):e029373. 10.1136/bmjopen-2019-029373 31748287 PMC6887068

[6] Braveman P . A health disparities perspective on obesity research. Prev Chronic Dis 2009;6(3):A91. 19527592 PMC2722388

[7] Schoenberg NE , Howell BM , Swanson M , Grosh C , Bardach S . Perspectives on healthy eating among Appalachian residents. J Rural Health 2013;29 (Suppl 1):s25–s34. 23944277 10.1111/jrh.12009 PMC3752844

[8] Olstad DL , Ancilotto R , Teychenne M , Minaker LM , Taber DR , Raine KD , Can targeted policies reduce obesity and improve obesity-related behaviours in socioeconomically disadvantaged populations? A systematic review. Obes Rev 2017;18(7):791–807. 10.1111/obr.12546 28434185

[9] Kumanyika S . Getting to equity in obesity prevention: a new framework. 2017. National Academy of Medicine. https://nam.edu/getting-to-equity-in-obesity-prevention-a-new-framework. Accessed November 17, 2020.

[10] Kumanyika SK . A framework for increasing equity impact in obesity prevention. Am J Public Health 2019;109(10):1350–7. 10.2105/AJPH.2019.305221 31415203 PMC6727309

[11] Centers for Disease Control and Prevention, Division of Nutrition, Physical Activity, and Obesity. High Obesity Program. https://www.cdc.gov/nccdphp/dnpao/state-local-programs/hop-1809/high-obesity-program-1809.html. 2020. Accessed June 15, 2020.

[12] US Census Bureau. QuickFacts, Martin County, Kentucky. https://www.census.gov/quickfacts/martincountykentucky. Published July 1, 2019. Accessed June 15, 2020.

[13] US Department of Agriculture, Economic Research Service. Food access research atlas. https://www.ers.usda.gov/data-products/food-access-research-atlas/go-to-the-atlas. Accessed August 27, 2020.

[14] Feeding America. Food insecurity in Martin County. https://map.feedingamerica.org/county/2018/overall/kentucky/county/martin. Accessed June 15, 2020.

[15] Grow Appalachia. Our mission. https://growappalachia.berea.edu/about/#. Accessed June 16, 2020.

[16] Chun Tie Y , Birks M , Francis K . Grounded theory research: a design framework for novice researchers. SAGE Open Med 2019;7:2050312118822927. 10.1177/2050312118822927 30637106 PMC6318722

[17] Jackson C , Perrett A , Descieux K . Agriculture and food system trends in the Appalachian region: 2007–2012. Appalachian Regional Commission; 2015. https://www.arc.gov/research/researchreportdetails.asp?REPORT_ID=119. Accessed June1, 2020.

[18] Marley BJ . The coal crisis in Appalachia: agrarian transformation, commodity frontiers and the geographies of capital. J Agrar Change 2016;16(2):225–54. 10.1111/joac.12104

[19] Perdue S , Hamer H . 2017 Census of Agriculture: Kentucky state and county data. US Department of Agriculture; 2019. https://www.nass.usda.gov/Publications/AgCensus/2017/Full_Report/Volume_1,_Chapter_2_County_Level/Kentucky/kyv1.pdf. Accessed June 15, 2020.

[20] Lichter DT , Garratt J , Marshall ML , Cardella M . Emerging patterns of population redistribution and migration in Appalachia. 2005. https://www.arc.gov/report/emerging-patterns-of-population-redistribution-and-migration-in-appalachia. Accessed November 17, 2020.

[21] Rossi J , Meyer AL , Knappage J . Beyond farmers markets: local foods opportunities in southeastern Kentucky’s retail and institutional industry. Community and Economic Development Initiative of Kentucky; 2018. https://cedik.ca.uky.edu/files/beyond_farmers_markets_final.pdf. Accessed November 17, 2020.

[22] Haskell J . Assessing the landscape of local food in Appalachia. Appalachian Regional Commission; 2012. https://www.arc.gov/images/programs/entrep/AssessingLandscapeofLocalFoodinAppalachia.pdf. Accessed May 1, 2020.

[23] Jilcott Pitts SB , Gustafson A , Wu Q , Leah Mayo M , Ward RK , McGuirt JT , Farmers’ market use is associated with fruit and vegetable consumption in diverse southern rural communities. Nutr J 2014;13(1):1. 10.1186/1475-2891-13-1 24405527 PMC3896848

[24] Sharaievska I , West S , Weddell M . The privilege of healthy eating: a qualitative study exploring the local food choices of low-income families from Appalachia. J Health Dispar Res Pract 2018;11(3).

[25] Behringer B , Friedell GH . Appalachia: where place matters in health. Prev Chronic Dis 2006;3(4):A113. 16978488 PMC1779277

[26] Appalachian Regional Commission. Poverty rates in Appalachia, 2013–2017. 2019. https://www.arc.gov/reports/custom_report.asp?REPORT_ID=77. Accessed June 17, 2020.

[27] Cafer A , Mann G , Ramachandran S , Kaiser M . National food affordability: a county-level analysis. Prev Chronic Dis 2018;15:E115. 10.5888/pcd15.180079 30240570 PMC6157262

[28] Webb SD . SOAR — the solution to the high prevalence of heart disease in Appalachian Kentucky. J Public Health (Berl) 2020. 10.1007/s10389-020-01248-5

[29] Feeding America. The impact of the coronavirus on food insecurity. 2020. https://www.feedingamericaaction.org/the-impact-of-coronavirus-on-food-insecurity. Accessed June 16, 2020.

[30] Miller WC , Rogalla D , Spencer D , Zia N , Griffith BN , Heinsberg HB . Community adaptations to an impending food desert in rural Appalachia, USA. Rural Remote Health 2016;16(4):3901. 27814451

